# The Impact of Social Media on Seeking Dermatological Care

**DOI:** 10.7759/cureus.49941

**Published:** 2023-12-05

**Authors:** Hanadi Alsatti, Sahal J Samarkandy, Dhai B Albogami, Rawan K Alrajhi, Raghad A Alahmadi, Rahaf Alturkistani, Shadi Alzahrani

**Affiliations:** 1 Dermatology, King Abdulaziz Medical City (KAMC), Jeddah, SAU; 2 Medicine, King Saud Bin Abdulaziz University for Health Sciences, Jeddah, SAU; 3 Medicine, King Abdulaziz Medical City (KAMC), Jeddah, SAU; 4 Oral and Maxillofacial Surgery, King Abdulaziz University, Jeddah, SAU

**Keywords:** social media, jeddah, filter, cosmetic, aesthetic

## Abstract

Introduction: Social media is used by more than half of the world's population. Social media is becoming more widely recognized as a significant factor when looking for healthcare services because of its capacity to affect an individual's decision. Therefore, we aim to investigate the influence of social media and the use of filters on seeking cosmetic dermatological procedures among the general population of Jeddah, Saudi Arabia.

Methods: This cross-sectional study utilized an online form for data collection. The questionnaire was developed by the authors after an extensive literature review. The data collection took place in October 2022 in Jeddah, Saudi Arabia. Participants were recruited using convenience and snowball sampling methods.

Results: A total of 1,031 responses were analyzed, with females representing the majority (85.2%, n = 878). About half (47.4%, n = 489) indicated a willingness to undergo an aesthetic procedure, 16.3% (n = 168) had already done one procedure at least, and 53.2% (n = 548) had a specific procedure to do in the future. Healthcare professionals were the most common source of information (53.9; n = 556), followed by social media (22.7%; n = 234). In the Snapchat application, 94.5% (n = 974) of the participants used face filters. Undergoing an aesthetic procedure showed statistically significant associations with age, gender, educational level, employment, and income.

Conclusion: Hiding skin lesions or acne was the most frequently reported reason for using photo editing apps or filters. While healthcare providers were the most frequently reported source of information, Snapchat influenced around 33% of the study’s participants to undergo an aesthetic procedure.

## Introduction

Social media use has rapidly increased over the last two decades. Facilitating communication between individuals or even large institutions has resulted in more than half of the child and adolescent population using social media [1, 2]. In fact, 41% of patients follow their current or potential physician on social media. It was found that having an online presence can influence patients' decisions to schedule an appointment by 43% [3]. Social media has a considerable effect on people's decisions regarding their dermatological care. As a result, social media ranked among the top three factors in buying decisions for skin care products and sixth among factors influencing the decision to have a cosmetic treatment. [3, 4] One key feature that most of the social media platforms offer is using photo editing features called “beauty filters." Platforms such as Snapchat, Instagram, and TikTok became more popular due to this feature. Since it allows individuals to enhance their appearance by altering the size of the nose or eyes, covering any skin blemishes, or even softening wrinkles [5], using filters may result in the desire to pursue cosmetic dermatology to achieve the effects of these filters in real life.

Although it is evident that social media has become the platform of choice for patients seeking care, there has been a substantial gap in the literature regarding its influence among dermatological patients, especially in Saudi Arabia. Hence, this study aimed to set an unprecedented report on the influence of social media and the usage of filters on seeking cosmetic dermatological procedures among the general population of Jeddah, Saudi Arabia.

## Materials and methods

Study design

This is a cross-sectional study that used an electronic questionnaire distributed via social media (Facebook (now known as Meta), Telegram, and Twitter (now known as X)). The authors developed the questionnaire according to available studies in the literature.

Study population

The study subjects included Saudi nationals aged 18 years and older who were living in Jeddah during the period of data collection. No exclusion criteria were applied. Participants were recruited using convenience and snowball sampling methods. We did not offer any incentives to them in return for their participation. The study was conducted in Jeddah, Saudi Arabia. Jeddah has an estimated population of 3.5 million [5]. Therefore, a sample of the population was calculated using a web-based calculator (Raosoft.com, Raosoft Inc., Seattle, WA) with a 5% margin of error, a confidence level of 95%, and an estimated 50% response distribution; the determined sample size was 385.

Data collection

We used a Google Form (Google Inc., Mountainview, CA) to collect data, and the corresponding link was distributed across various social media platforms, including WhatsApp, Snapchat, and Twitter (now known as X). The questionnaire underwent a preliminary pilot phase before the official data collection began. During this stage, each author was tasked with gathering feedback from five different people. The purpose of this feedback was to assess both the time required to complete the questionnaire and the clarity of its wording. It should be noted that the responses obtained during the pilot phase were not included in the final analysis. Because we used snowball and convenience sampling methods, we were unable to assess the non-response rate.

The studied variables of the questionnaire were divided into three sections: patient demographics, social media use, and history and intentions regarding cosmetic procedures. The questionnaire had 17 questions developed by the authors after an extensive literature review of similar studies (Appendix A). It was pilot-tested on a random sample of 10 patients. The decision to have an aesthetic procedure, among other variables, was assessed using yes-or-no questions. Likert scale and checkbox questions were also used for data collection.

Statistical analysis

The analysis was done using IBM SPSS software version 27.0 (IBM Corp., Armonk, NY). During data sorting and coding, 24 responses were excluded due to participants' refusal to provide consent, and 375 responses were removed as they were not residents of Jeddah city. Frequency tables and proportions were used to present the variables. For statistical associations, a chi-square test was used with a P-value<0.05 as the level of significance.

Ethical considerations

This study was approved by the institutional review board of the King Abdullah International Medical Research Center, Jeddah, Saudi Arabia, on September 28, 2022 (approval number: SP22J/138/09). A consent statement on the cover page of the questionnaire was added to explain the nature and objectives of the study. The participants were also informed of the study benefits and the estimated period needed to fill out the survey. No personal identifiers were used to assure anonymity. Only the investigators had access to the data. The data were secured and used for research purposes only.

## Results

A total of 1,031 responses were analyzed. The age group of 18-24 represented 35.3% (n = 364), with females representing the majority at 85.2% (n = 878). More than half (54.3%, n = 560) of the participants had a bachelor's degree, and employed participants represented 45.5% (n = 469), with half of the participants (49.3%, n = 508) having a monthly income less than 5,000 Saudi riyals (SAR). The sociodemographic characteristics of the participants are shown in Table 1.

**Table 1:** The sociodemographic characteristics of the participants (n = 1031) The data are presented in the form of numbers (percentages). SAR: Saudi Riyal

Studied variables		N	%
Age (in years)	18-24	364	35.3%
	25-34	358	34.7%
	>34	309	30.0%
Gender	Male	153	14.8%
	Female	878	85.2%
Marital status	Single	527	51.1%
	Married	504	48.9%
Highest level of education	Less than a high school degree	27	2.6%
	High school	154	14.9%
	In college, no degree	216	21.0%
	Bachelor's degree	560	54.3%
	Higher education	74	7.2%
Employment	Unable to work	13	1.3%
	Unemployed	214	20.8%
	Student	282	27.4%
	Employed	469	45.5%
	Retired	53	5.1%
Monthly income	<5000 SAR	508	49.3%
	5000-10000 SAR	278	27.0%
	11000-15000 SAR	122	11.8%
	16000-20000 SAR	72	7.0%
	21000-50000 SAR	51	4.9%

Of the total, 47.4% (n = 489) indicated a willingness to undergo an aesthetic procedure, and 16.3% (n = 168) have already done one at least. Two-thirds (67.1%, n = 692) indicated that they are aware of cosmetic procedure complications, with a similar percentage (64.7%, n = 667) indicating that the price of the procedure can influence their decision. The participants were also asked about their usual source of information regarding cosmetic procedures. Healthcare professionals were the most frequent source, as indicated by 53.9% (n = 556); social media was indicated by 22.7% (n = 234). The sources of information are tabulated in Table 2.

**Table 2:** The participants' sources of information about cosmetic procedures The data are presented in the form of numbers (percentages).

Source of information	Yes		No	
	N	%	N	%
Medical websites	493	47.8%	538	52.2%
Family and friends' experiences	417	40.4%	614	59.6%
Healthcare professionals	545	52.9%	486	47.1%
Social media	234	22.7%	797	77.3%
Others	5	0.5%	1026	99.5%

**Figure 1:**
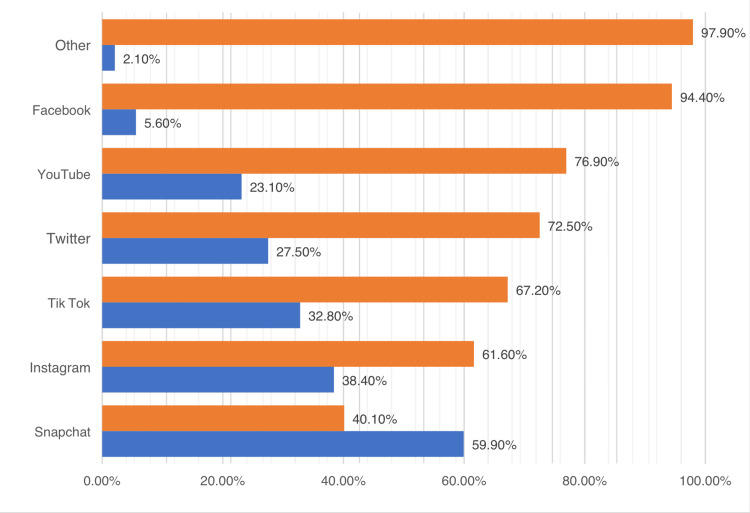
The primary social media platforms utilized by the participants

Snapchat was chosen as the most popular social media application among 59.9% (n = 614). Figure 1 lists the social media platforms that participants utilized most frequently.

**Figure 2:**
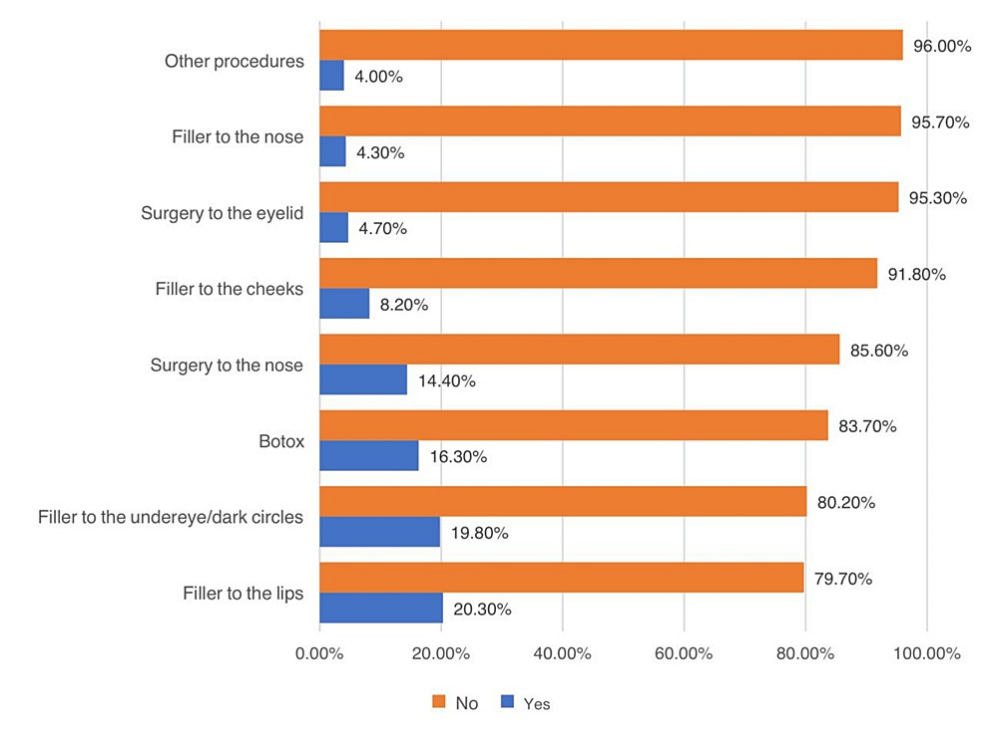
Types of procedures the participants intend to undertake in the future

While using Snapchat, 94.5% (n = 974) of the participants used face filters. The reasons for using the filters were to change the shape of the eye, nose, or lips (24%, n = 247), to hide skin lesions and acne (59.3%, n = 611), to hide dark circles (38.2%, n = 394), to soften wrinkles (13%, n = 134), and other reasons (1.8%, n = 19). Snapchat also enhanced thoughts of having facial cosmetic changes among 34.9% (n = 360). More than half (53.2%, n = 548) indicated having a specific procedure that they would like to undergo in the future. Of those who intended specific procedures, the following were chosen: filler to the lips (35.2%, n = 363), filler to the undereye (34.6%, n = 357), filler to the cheeks (14.9%, n = 154), filler to the nose (6.9%, n = 71), Botox (29.3%, n = 302), nasal surgery (25.5%, n = 263), eyelid surgery (8%, n = 82), and other procedures (6.2%, n = 64). The intended future procedures are demonstrated by the total sample size in Figure 2.

The question "Have you ever had an aesthetic procedure?" was tested for statistically significant associations with demographic variables; age, gender, educational level, employment, and monthly income showed statistically significant associations. The detailed proportions and P-values are shown in Table 3.

**Table 3:** Cross-tabulation showing the association between having aesthetic procedures and sociodemographic variables SAR: Saudi Riyal; P<0.05 is considered significant; Chi-square test of significance; one USD = 3.75 SAR

Studied variables		Have you ever had an aesthetic procedure?				
		Yes		No		P-value
		N	%	N	%	
Age (in years)	18-24	41	11.3%	323	88.7%	0.001
	25-34	76	21.2%	282	78.8%	
	>34	51	16.5%	258	83.5%	
Gender	Male	14	9.2%	139	90.8%	0.010
	Female	154	17.5%	724	82.5%	
Marital status	Single	77	14.6%	450	85.4%	0.134
	Married	91	18.1%	413	81.9%	
Highest level of education	Less than high school	5	18.5%	22	81.5%	<0.001
	High school	21	13.6%	133	86.4%	
	In college, no degree	25	11.6%	191	88.4%	
	Bachelor's degree	93	16.6%	467	83.4%	
	Higher education	24	32.4%	50	67.6%	
Employment	Unable to work	0	0.0%	13	100.0%	<0.001
	Unemployed	27	12.6%	187	87.4%	
	Student	29	10.3%	253	89.7%	
	Employed	101	21.5%	368	78.5%	
	Retired	11	20.8%	42	79.2%	
Monthly income	<5000 SAR	56	11.0%	452	89.0%	<0.001
	5000-10000 SAR	54	19.4%	224	80.6%	
	11000-15000 SAR	25	20.5%	97	79.5%	
	16000-20000 SAR	24	33.3%	48	66.7%	
	21000-50000 SAR	9	17.6%	42	82.4%	

Demographic variables were also tested for statistical significance, with the influence of Snapchat on thoughts about facial cosmetic procedures. In this regard, age, gender, and employment showed statistically significant associations, which are presented in Table 4.

**Table 4:** Cross-tabulation showing the association between the influence of Snapchat on cosmetic procedure thoughts and sociodemographic variables SAR: Saudi Riyal; P<0.05 is considered significant; Chi-square test of significance; one USD = 3.75 SAR

Studied variables		Did Snapchat make you think of having some facial changes?				
		Yes		No		P-value
		N	%	N	%	
Age (in years)	18-24	133	36.5%	231	63.5%	0.034
	25-34	137	38.3%	221	61.7%	
	>34	90	29.1%	219	70.9%	
Gender	Male	41	26.8%	112	73.2%	0.022
	Female	319	36.3%	559	63.7%	
Marital status	Single	183	34.7%	344	65.3%	0.894
	Married	177	35.1%	327	64.9%	
Highest level of education	Less than high school	13	48.1%	14	51.9%	0.623
	High school	53	34.4%	101	65.6%	
	In college, no degree	76	35.2%	140	64.8%	
	Bachelor's degree	190	33.9%	370	66.1%	
	Higher education	28	37.8%	46	62.2%	
Employment	Unable to work	5	38.5%	8	61.5%	<0.001
	Unemployed	60	28.0%	154	72.0%	
	Student	92	32.6%	190	67.4%	
	Employed	194	41.4%	275	58.6%	
	Retired	9	17.0%	44	83.0%	
Monthly income	<5000 SAR	173	34.1%	335	65.9%	0.793
	5000-10000 SAR	97	34.9%	181	65.1%	
	11000-15000 SAR	43	35.2%	79	64.8%	
	16000-20000 SAR	25	34.7%	47	65.3%	
	21000-50000 SAR	22	43.1%	29	56.9%	

Similarly, undergoing a specific procedure intended for the future was also tested for significance, in which only gender showed statistical significance. The related results are in Table 5.

**Table 5:** Cross-tabulation showing the association between planning on a specific procedure in the future and sociodemographic variables SAR: Saudi Riyal; P<0.05 is considered significant; Chi-square test of significance

		Is there any specific procedure you would like to do in the future?				
		Yes		No		P-value
		N	%	N	%	
Age (in years)	18-24	193	53.0%	171	47.0%	0.170
	25-34	179	50.0%	179	50.0%	
	>34	177	57.3%	132	42.7%	
Gender	Male	57	37.3%	96	62.7%	<0.001
	Female	492	56.0%	386	44.0%	
Marital status	Single	281	53.3%	246	46.7%	0.963
	Married	268	53.2%	236	46.8%	
Highest level of education	Less than high school	13	48.1%	14	51.9%	0.493
	High school	77	50.0%	77	50.0%	
	In college, no degree	117	54.2%	99	45.8%	
	Bachelor's degree	296	52.9%	264	47.1%	
	Higher education	46	62.2%	28	37.8%	
Employment	Unable to work	6	46.2%	7	53.8%	0.663
	Unemployed	107	50.0%	107	50.0%	
	Student	159	56.4%	123	43.6%	
	Employed	250	53.3%	219	46.7%	
	Retired	27	50.9%	26	49.1%	
Monthly income	<5000 SAR	262	51.6%	246	48.4%	0.423
	5000-10000 SAR	155	55.8%	123	44.2%	
	11000-15000 SAR	64	52.5%	58	47.5%	
	16000-20000 SAR	44	61.1%	28	38.9%	
	21000-50000 SAR	24	47.1%	27	52.9%	

## Discussion

Social media and its platforms are extremely popular nowadays, and they are getting progressively more attention over time, especially among the younger population. Sometimes people use them as their primary source of information; in fact, about 83% of the young population seeks medical information or attention over the Internet [6-10]. The results of the current study, which involved around 1,000 participants, showed that Snapchat was the most commonly used social media platform (60%). In contrast, the most commonly used social media platform is Facebook (now called Meta), followed by YouTube, as concluded in another study [6]. As per the aforementioned study survey results, participants are using social media platforms for around three hours daily on average [6, 11].

Photo editing applications or features, which have already been implemented on social media platforms, are used most of the time to edit imperfections noted in some of the pictures before posting them [1]. As per the 2017 annual American Academy of Facial Plastic and Reconstructive Surgery survey, around 55% of surgeons who participated reported that a very common reason among patients who are requesting surgery was to improve their appearance in selfies [1]. Participants in the current study reported using filters on Snapchat mostly to hide skin lesions and acne (59.3%), followed by hiding dark circles (38.2%), and to change the shape of their lips, nose, or eyes (24%). Moreover, another population-based survey study that involved around 250 participants regarding the effects of photo editing on seeking dermatological care revealed that 88.7% of participants edited their photos before posting them on social media, the most commonly reported reason for which was editing a skin lesion, comprising around 45% of all reasons reported in the study [12]. Another survey-based study involving 550 participants revealed that hiding skin lesions or skin pigmentation was the most commonly reported reason for editing a photo before posting it, which is in line with the results mentioned by the current study as well as the study done by Agrawal et al. [6]

As for the sources of information regarding cosmetic or dermatological procedures noted by participants in this study, healthcare professionals were the most common source of information, comprising 52.9%, followed by medical websites (47.8%), family and friends (40.4%), and finally social media (22.7%). Whereas, per the results of the study done by Agrawal et al., participants sought information and advice regarding cosmetic care or dermatological care most commonly through beauty influencers over the internet or social media (38.2%), followed by a dermatologist (37.4%), and finally family and friends (22%) [6]. Furthermore, another study reported that potential influential sources to seek cosmetic care were as follows: one’s own desire (44%), a physician (23.5%), and family and friends (15.3%) [13]. As concluded based on our results, around 50% of all participants demonstrated a willingness to undergo a cosmetic or dermatological procedure, and 16.3% of all participants had already undergone one. In comparison, it was reported by Agrawal et al. that about 25% of their participants had already visited a dermatologist for cosmetics or skincare, and around 70% of the participants were willing to visit a dermatologist in the future [6].

Snapchat motivated around one-third of this study’s participants to undergo facial cosmetic procedures, and approximately 50% of them determined a specific procedure that they would like to undergo in the future. As per Montemurro et al., social media has greatly impacted people’s decisions regarding aesthetic breast augmentation, as around 90% of people searched for information online regarding this aesthetic procedure, and it was also noted by approximately 50% of surgeons that their consultations were affected by social media [14].

There were multiple noteworthy associations between sociodemographic factors and having an aesthetic procedure, based on this study’s results. We found that participants aged between 25 and 34 were found to be the most common age group to have already undergone an aesthetic procedure, and this is in line with the age groups mentioned in the literature [6]. Females who have undergone an aesthetic procedure are more than males, as are those with higher educational levels. Moreover, the higher the monthly income, the more likely participants are to do the procedure, and it has also been found to be one of the most commonly reported limitations of doing a cosmetic or dermatological procedure. In concordance with our results, Sobanko et al. concluded in their prospective study involving 72 patients regarding motivations for seeking minimally invasive cosmetic procedures that the main limitation for undergoing an aesthetic procedure is the financial aspect and the high costs associated with it, which was supported by another study as well [13, 15].

Strengths and limitations

This study has several limitations. The aforementioned factors include the potential for sampling bias resulting from the non-random sampling technique, the reliance on self-reported data, which could potentially introduce biases related to recall and social desirability, limited generalizability to populations beyond the study settings, the inability of the cross-sectional design to establish causality, the possibility of non-response bias among non-participants, and a single assessment point that may not be able to capture changing attitudes.

## Conclusions

Social media has been exponentially growing in popularity and has been an essential platform for sharing information and experiences. A lot of people are motivated to make a decision or make a change in their lives based on social media influencers. As for the reported sources of information and motivation, the current study revealed the most common source of information among participants to be healthcare providers, whereas another study revealed social media influencers to be the most common source. This study evaluated the impact of social media on influencing people to seek dermatological or cosmetic care. According to our study, Snapchat was found to be the most commonly used social media platform; however, other studies concluded that Facebook (now Meta) was the most common. Furthermore, the most frequently reported reason for using photo editing applications or filters implemented in social media platforms was to hide skin lesions or acne, as evident based on this study’s results as well as those of other studies. Snapchat influenced around 33% of this study’s participants to undergo an aesthetic procedure, and half of them have already decided which specific procedure they would like to undergo, proving that social media platforms indeed greatly influence people to seek skin and cosmetic care.

## Figures and Tables

**Figure 3:**
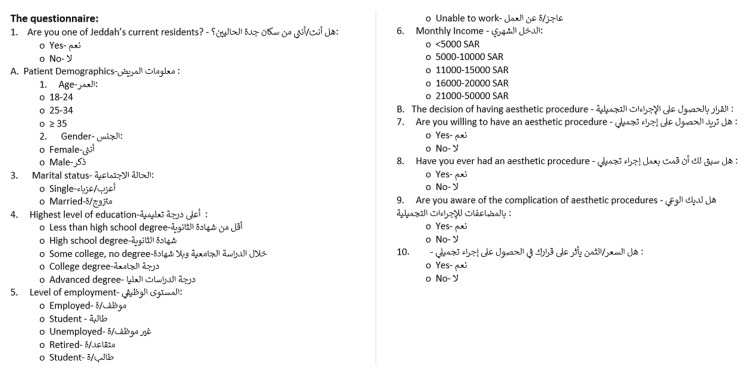
Parts A and B of the questionnaire sent to the participants

**Figure 4:**
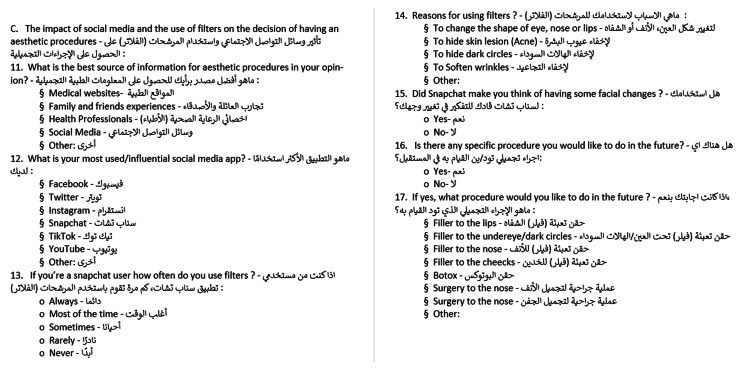
Part C of the questionnaire sent to the participants

## Appendices

Appendix A
